# Identification and characteristic analysis of enhancers across 13 major cancer types

**DOI:** 10.1093/pcmedi/pbab019

**Published:** 2021-08-02

**Authors:** Mingming Qian, Wenzhu Wang, Yana Zhang, Yi Zhao, Huige Quan, Yuting Chen, Xinyue Dai, Zhiyun Guo

**Affiliations:** School of Life Sciences and Engineering, Southwest Jiaotong University, Chengdu 610031, China; School of Life Sciences and Engineering, Southwest Jiaotong University, Chengdu 610031, China; School of Life Sciences and Engineering, Southwest Jiaotong University, Chengdu 610031, China; School of Life Sciences and Engineering, Southwest Jiaotong University, Chengdu 610031, China; School of Life Sciences and Engineering, Southwest Jiaotong University, Chengdu 610031, China; School of Life Sciences and Engineering, Southwest Jiaotong University, Chengdu 610031, China; School of Life Sciences and Engineering, Southwest Jiaotong University, Chengdu 610031, China; School of Life Sciences and Engineering, Southwest Jiaotong University, Chengdu 610031, China

**Keywords:** cancer, TCGA, enhancers, transcription factors, single nucleotide polymorphisms (SNPs)

## Abstract

Enhancers are often mutated and dysregulated in various diseases such as cancer. By integrating the function annotation of the mammalian genome (FANTOM) enhancers expression profiles and RNA-seq data from The Cancer Genome Atlas (TCGA) of 13 cancers and their corresponding *para*-cancerous tissues, we systematically identified a total of 4702 significantly differentially expressed (DE) enhancers. Furthermore, a total of 1036 DE genes regulated by DE enhancers were identified. It was found that in these 13 cancers, most (61.13%) enhancers were ubiquitously expressed, whereas DE enhancers were more likely to be tissue-specific expressed, and the DE genes regulated by DE enhancers were significantly enriched in cancer-related pathways. Finally, it was manifested that 74 single nucleotide polymorphisms (SNPs) were located in 37 DE enhancers, and these SNPs affected the gain and loss of functional transcription factor binding sites of 758 transcription factors, which were shown to be highly correlated with tumorigenesis and development.

## Introduction

Enhancers is a class of *cis*-acting elements in the genome of eukaryotes, which can positively regulate gene expression; dysregulation of enhancers can cause a variety of diseases, such as cancer.^1,2^ Transcriptomics and genomics studies have found that an active enhancer can be used as a transcription unit to transcribe eRNA (enhancer RNA).^3^ In cell signaling and transcriptional regulation, the expression level of eRNA is highly correlated with the activity of functional enhancer.^4^ In addition, mutations in the enhancers may affect the gain and loss of transcription factor binding sites (TFBS) on the enhancer.^5^ These studies suggest that enhancer mutations may be involved in biological processes and signaling pathways of cancer.

In order to analyze and identify the relationship between dysregulated enhancers and tumorigenesis, we identified many differentially expressed (DE) enhancers in these 13 cancers and matched *para*-cancerous tissues of The Cancer Genome Atlas (TCGA) using multi-omics approaches. Then, many DE genes were obtained that were coexpression-regulated by DE enhancers in cancers. Finally, a series of single nucleotide polymorphisms (SNPs), which may affect the gain and loss of TFBS, were identified, and transcription factors (TFs) located in these TFBS are highly correlated with cancer, suggesting that they could be used as potential targets for future cancer drugs.

## Materials and methods

### Identification of differentially expressed enhancers and genes

The enhancer region (hg19) and expression profile data (reads per kilobase per million mapped reads) of 13 cancers and matched *para*-cancerous tissues (5727 cancer samples and 619 normal samples) were obtained from a previous study.^6^ The 65 423 FANTOM enhancers capable of transcribing eRNA were re-annotated. They then removed those enhancers that overlapped with known genes or intron regions. RNA-Seq data of 13 cancers and *para*-cancerous tissues (613 cancer samples and 609 normal samples) were downloaded from TCGA (https://portal.gdc.cancer.gov/). The enhancers/genes with expression in at least 10% of cancers/normal tissue samples were identified as candidate enhancers/genes. Significantly differentially expressed enhancers/genes between cancers and *para*-cancerous tissues were determined according to the following criterion (|log_2_FC|≥1, *q*-value < 0.05, *t*-test). In the same cancer, for each enhancer (gene as well), the average of its expression across samples was used to calculate log2FC and *t*-test *P*-values.

### Identification of DE enhancers–DE gene interactions

The transcription start sites (TSSs) of genes were retrieved by processing GENCODE annotations v19, and the 0.5 kb upstream and 1 kb downstream from TSS were used as promoter regions.^7^ If a gene promoter intersected with the region 100 kb upstream and downstream from the enhancer center, the gene was considered as a candidate target gene of the enhancer.^8^ Considering that the expression of both enhancers and genes differed in different tissues, we processed the RNA-seq data from different samples separately. The Spearman correlation between enhancer and gene expression was calculated and screened with corr ≥ 0.3 and *q*-value < 0.05 as the threshold.

### Kyoto Encyclopedia of Genes and Genomes (KEGG) enrichment analysis of target genes

In order to further understand the biological mechanism of the DE enhancer–DE gene interactions, KEGG functional enrichment analysis of the target genes of DE enhancers was performed using the "clusterProfiler" package^9^ (R 3.6.0). The threshold of a statistically significant difference was *q*-value < 0.05.

### Identification of enhancers SNPs and analysis of TFBS

The SNPs data (GRCh38) of 13 TCGA cancers was obtained from the ncRNA-eQTLs database,^10^ including SNP loci, rs ID, etc. First, the enhancer region was changed from hg19 to GRCh38 using the LiftOver tool of UCSC.^11^ Then, the enhancer region was extended to 0.5 kb upstream and 0.5 kb downstream from the enhancer boundary, and the cancer SNPs' loci were mapped to the DE enhancers region to obtain enhancer SNPs. By using the atSNP tool,^12^ the TFs affected by SNPs were identified, and the effects of SNPs on TFBS were analyzed. Gene Expressiob Profiling Interactive Analysis^13^ was used to identify the survival curve of genes in cancer, with *q*-value < 0.05 as the threshold.

## Results and discussion

### Differential expression analysis of enhancers in 13 cancers

**Figure 1.**
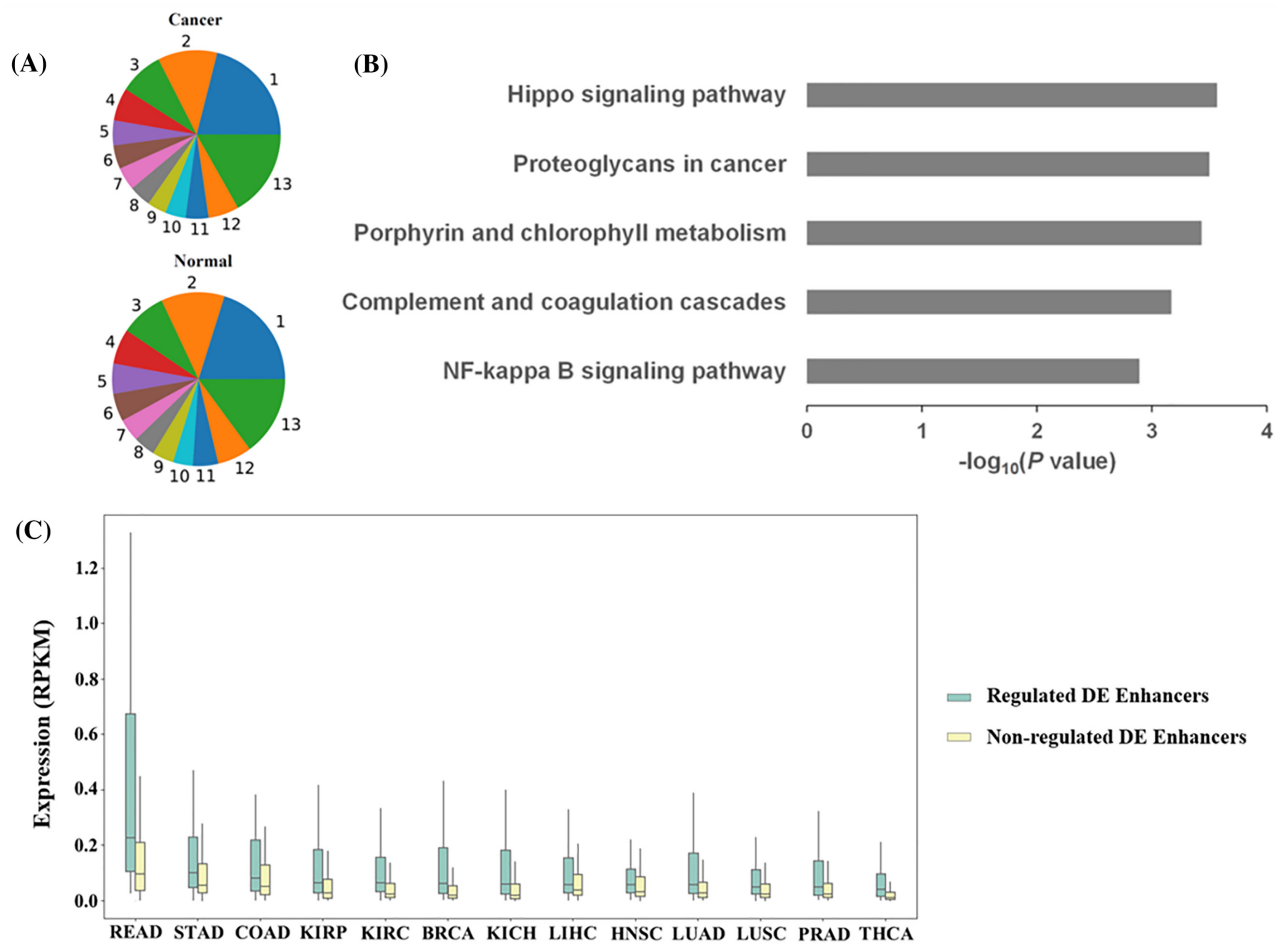
Differential expression analysis of enhancers in 13 cancers. (A) The distribution of enhancers across 13 cancers or *para*-cancerous tissues. The numbers and color blocks represent the proportion of all enhancers expressed in different numbers of cancers or *para*-cancerous tissues. (B) Kyoto Encyclopedia of Genes and Genomes (KEGG) analysis of differentially expressed (DE) genes that were participating in the coexpression regulation with DE enhancers. The gray bars indicate the -log_10_(*P* value) of these KEGG pathways. (C) Signature difference between regulated DE enhancers and nonregulated DE enhancers. Comparison of the expression level of regulated DE enhancers and nonregulated DE enhancers in 13 types of cancers, *P*-value < 0.05.

The expression profiles of the enhancers were analyzed in 13 cancers and matched *para*-cancerous tissues, yielding in 10 639 and 10 039 enhancers, respectively. Based on the expression in 13 cancers and *para*-cancerous tissues, the enhancers were divided into three categories: tissue-specific enhancers (enhancers expressed in only one tissue, see blue block labeled 1 in Fig. 1A), ubiquitously expressed enhancers (enhancers expressed in all tissues, see green block labeled 13 on Fig. 1A), and other enhancers (see color blocks labeled 2–12 on Fig. 1A). Statistics analysis on the types of all enhancers revealed that the distribution of enhancers was obviously tissue-specific and ubiquitously expressed, whether in the cancers or *para*-cancerous tissues (Fig. 1A). In a certain type of cancer or normal tissue, the ubiquitously expressed and tissue-specific enhancers occupied the largest and smallest proportion, respectively (Supplementary Fig. S1). These results might indicate that most enhancers participated in the tissue regulatory network as widely regulatory factors, apart from a few tissue-specific enhancers which were responsible for tissue identification and development.

### Target genes of DE enhancers are significantly associated with cancer

Based on differential expression, a total of 4702 significantly DE enhancers were identified in these 13 cancers (|log_2_FC|≥1, *q*-value < 0.05), among which 1646 and 1589 enhancers were differentially upregulated or downregulated, respectively (Supplementary Table S1). The function of enhancers is mainly achieved by regulating the downstream target genes. Finally, a total of 14 461 DE genes were obtained, among which 5357 and 2933 enhancers were differentially upregulated or downregulated, respectively (Supplementary Table S1). Although Hi-C data had been used for identification of enhancer target genes, due to the acquisition limitaion and low resolution of Hi-C data, our identified enhancer targets refer to a previous study.^6^ By integrating distance analysis (within the enhancer center ± 100 kb region) and coexpression analysis (Spearman correlation corr ≥ 0.3 and *q*-value < 0.05), a total of 1036 DE genes were identified in these 13 cancers; these genes are potentially subject to regulation by DE enhancers (Supplementary Table S2). We divide DE enhancers into two categories: DE enhancers that regulate DE genes (regulated DE enhancers) and DE enhancers that do not regulate DE genes (nonregulated DE enhancers). It was found that most (3662 of 4540/80.66%) of the DE enhancers that regulated DE genes were ubiquitously expressed (Supplementary Fig. S2). This result suggested that dysregulated enhancers were mainly involved in the fundamental biological process of tumors. In order to explore the association between cancer and target genes of DE enhancers, KEGG enrichment analysis was performed on the target genes. The DE genes that were regulated by DE enhancers were significantly enriched in cancer-related pathways such as the hippo signaling pathway, proteoglycans cancer, and other cancer-related KEGG pathways (Fig. 1B), indicating that these DE enhancers may also play an important role in cancer. We also compared the enrichment pathways of DE genes that are not regulated by DE enhancers (Supplementary Fig. S4), and we could see that they are enriched in pathways that are almost unrelated to cancer. We next investigated the expression levels of target genes regulated by the DE enhancer and those not regulated by the DE enhancer, and found that the expression level of regulated DE enhancers was significantly higher than that of nonregulated DE enhancers in these 13 cancers (Fig. 1C, *P*-value < 0.05).

### The identification of DE enhancers sequence variants and TF dysregulation affected by these variants

**Figure 2.**
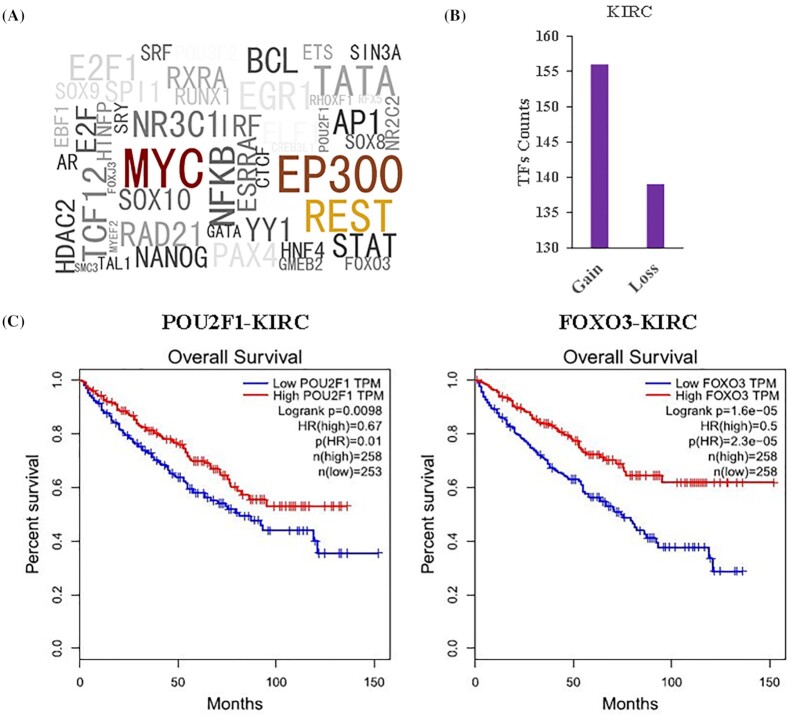
The gain and loss of functional transcription factor binding sites (TFBS) and survival analysis of transcription factors (TFs). (A) The top 50 TFs that were most affected by the SNPs, which may relate to enhancer activity. The larger the logo of the TF name, the more SNPs influence this TF. (B) The gain and loss of functional TFBS of 241 TFs influenced by SNPs located in enhancer chr20:59164329–59165752. (C) The survival analysis of the transcription factors POU2F1 and FOXO3, *q*-value < 0.05.

Previous studies have proved that mutations in enhancers are highly related to the development of cancer, and SNPs could participate in the regulation of multiple biological processes and trigger multiple diseases by affecting the gain and loss of TFBS.^14^ To further investigate this, we identified a total of 74 SNPs that were located in 37 regulated DE enhancers in 10 cancers through extracting the ncRNA-eQTL database (Supplementary Table S3). By using the atSNP tool, 758 TFs which had gain or loss of functional TFBS influenced by these SNPs were identified, and we found that most of these TFs were well-known cancer-related genes such as *MYC, EP300*, and *REST* (Fig. 2A, Supplementary Table S4). In particular, as a histone acetyltransferase, *EP300* regulates transcription by chromatin remodeling and plays an important role in cell proliferation, transformation, and differentiation. It has become a key TF for identifying enhancers.^15^ These results indicate that mutations in enhancers could interfere with the role of enhancers in cancer by affecting the binding of cancer-related TFs to enhancers.

We noticed that the highest proportion of SNPs (28.4%) were found in kidney renal clear cell carcinoma (KIRC) compared with other cancers. A total of 21 SNPs were identified in 15 enhancers, which affected 511 TFs (Supplementary Table S3). The enhancer located in chr20:59164329–59165752 contained five SNPs (rs73306874, rs6026739, rs6026740, rs6026742, rs73306876) that affected 241 TFs gaining or losing TF binding sites on this enhancer (Fig. 2B). The top three TFs most affected by SNPs were SOX9 (affected by SNP rs73306874, rs6026739, rs6026740, rs73306876), POU2F1 (effected by rs73306874, rs6026739, rs73306876), and FOXO3 (effected by rs73306874, rs6026740, rs73306876). Survival analysis showed that the expression of POU2F1 and FOXO3 was significantly related to the survival time of patients with KIRC (Fig. 2C). Although the correlation between SOX9 and the survival time of KIRC patients was not significant, a previous study has shown that SOX9 could inhibit cell proliferation and invasion of renal cell carcinoma, in the way of being targeted by microRNA-138.^16^ In summary, these SNPs influence the survival time of cancer patients by affecting TF binding to enhancers and can be used as potential enhancer-targeted drugs target sites.

## Conclusions

In summary, we integrated RNA-seq data from FANTOM enhancer expression profiles and TCGA to systematically identify a total of 4702 significantly differentially expressed enhancers in 13 cancers and matched *para*-cancerous tissues. In addition, a total of 1036 DE genes regulated by DE enhancers were identified by integration distance and coexpression analysis. We found that DE enhancers were more likely to be tissue-specific in their expression and that DE genes regulated by DE enhancers were significantly enriched in cancer-related pathways. Finally, the results showed that 74 SNPs located in 37 DE enhancers and affected the gain and loss of functional TFBS of 758 transcription factors, which had been proved to be highly related to the occurrence and development of tumors. Taken together, these results provide informative data and methods of dysregulated enhancers mutations for future research in cancer treatment.

## Supplementary Material

pbab019_Supplemental_Files
