# Supplementary material for: Identification and characteristic analysis of enhancers across 13 major cancer types
Source: Precis Clin Med. 2021 Aug 2;4(3):204–8. doi: 10.1093/pcmedi/pbab019 (PMC8982554; doi:10.1093/pcmedi/pbab019)
Supplement: pbab019_Supplemental_Files [file pbab019_supplemental_files.zip › Supplementary_Figures.pdf]

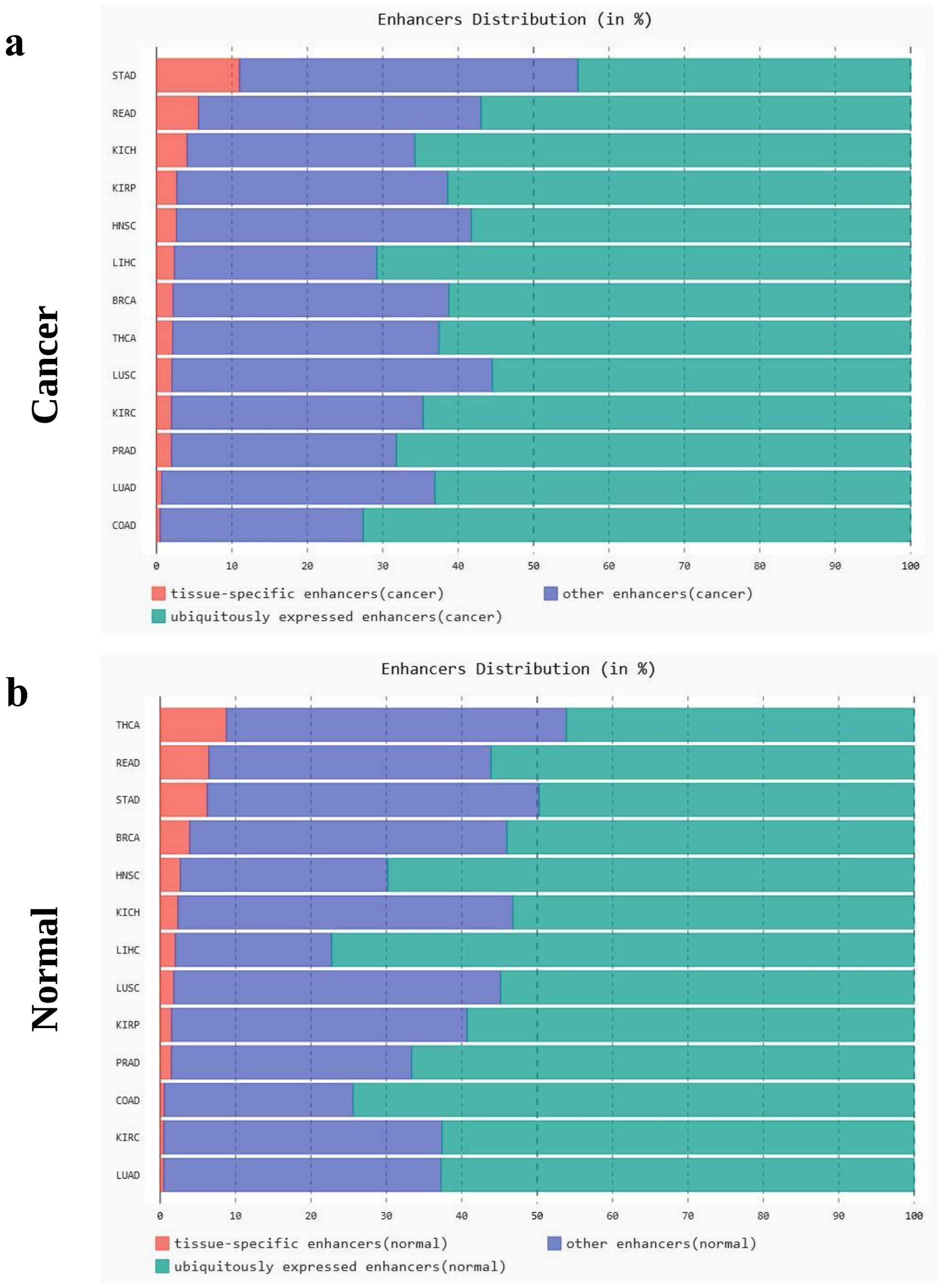

Supplementary Figure S1. Statistic on proportion of three types of enhancers. **a** Percentage of tissue-specific enhancers, ubiquitously expressed enhancers, other enhancers in each cancer tissue. **b** Percentage of tissue-specific enhancers, ubiquitously expressed enhancers, other enhancers in each normal tissue

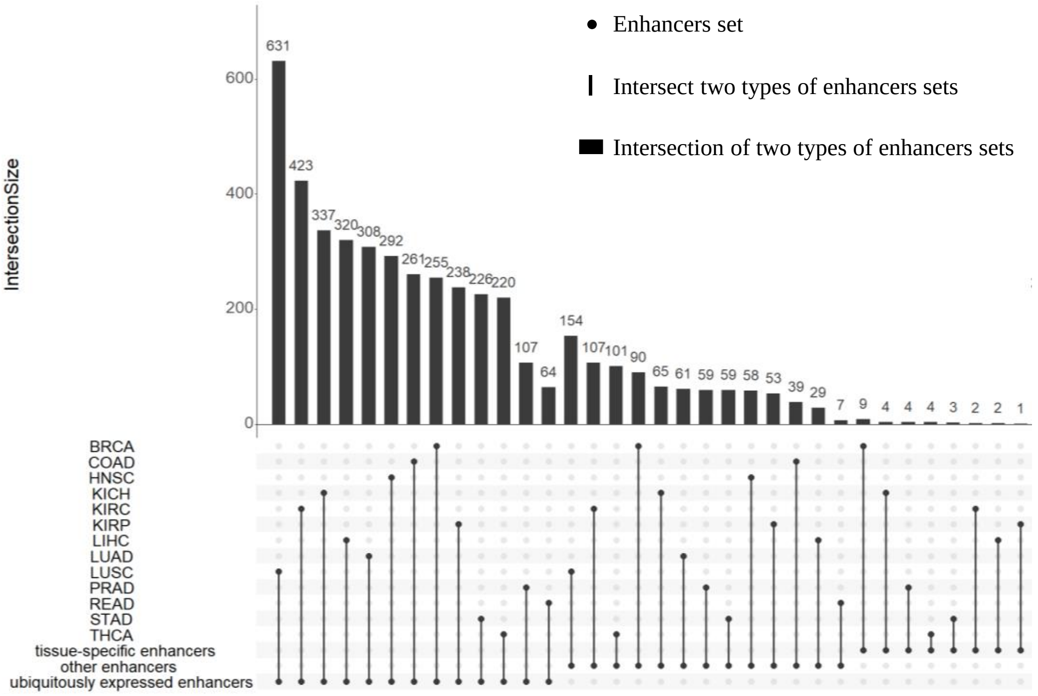

Supplementary Figure S2. The intersection of regulated DE enhancers and three types of enhancers in each cancer. regulated DE expressed enhancers: the differentially enhancers that regulated differentially expressed genes. Three types of enhancers: tissue-specific enhancers, ubiquitously expressed enhancers, and other enhancers. The number on the bar chart represents the intersection number of the corresponding two kinds of enhancers

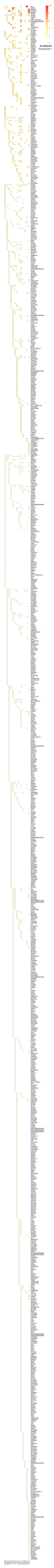

Supplementary Figure S3. The number of DE enhancers which regulated DE genes. Note: The number in the box indicates the number of DE enhancers that regulated this gene in the cancer. The redder the color, the greater the number of DE enhancers

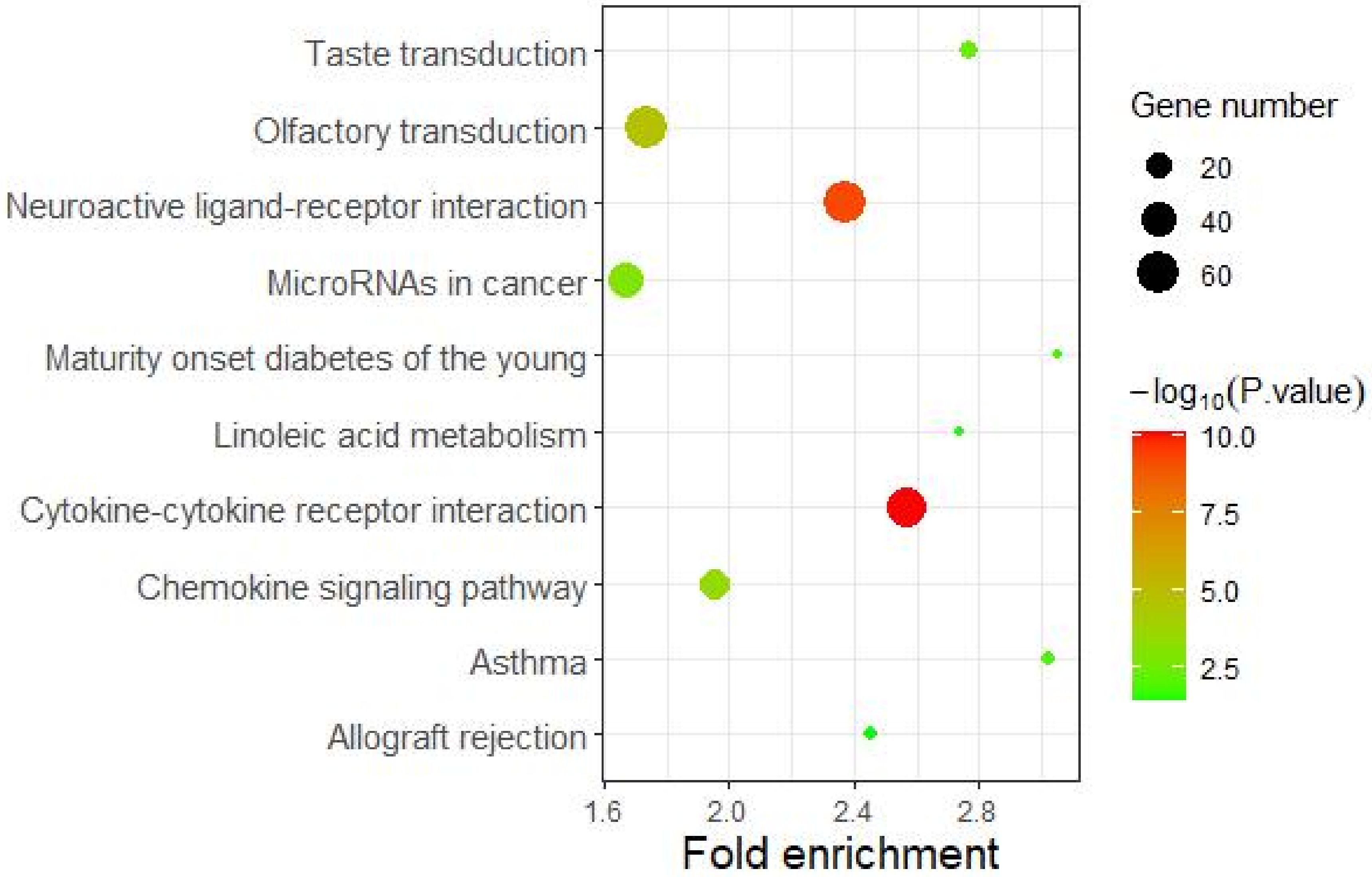

Supplementary Figure S4. KEGG analysis of differentially expressed genes which were not participating in the co-expression regulation with differentially expressed enhancers.
